# Supplementary material for: Capturing colloidal nano- and microplastics with plant-based nanocellulose networks
Source: Nat Commun. 2022 Apr 5;13:1814. doi: 10.1038/s41467-022-29446-7 (PMC8983699; doi:10.1038/s41467-022-29446-7)
Supplement: Supplementary file 1 — Supplementary Information [file 41467_2022_29446_MOESM1_ESM.pdf]

SUPPLEMENTARY INFORMATION FOR

Capturing colloidal nano- and microplastics with plant-based nanocellulose networks

Ilona Leppänen[1], Timo Lappalainen[1], Tia Lohtander[1,2], Christopher Jonkergouw[3], Suvi Arola[1*] and Tekla Tammelin[1*]

This file includes:

Supplementary Figures 1 -15

Supplementary Tables 1 - 5

Supplementary Figures

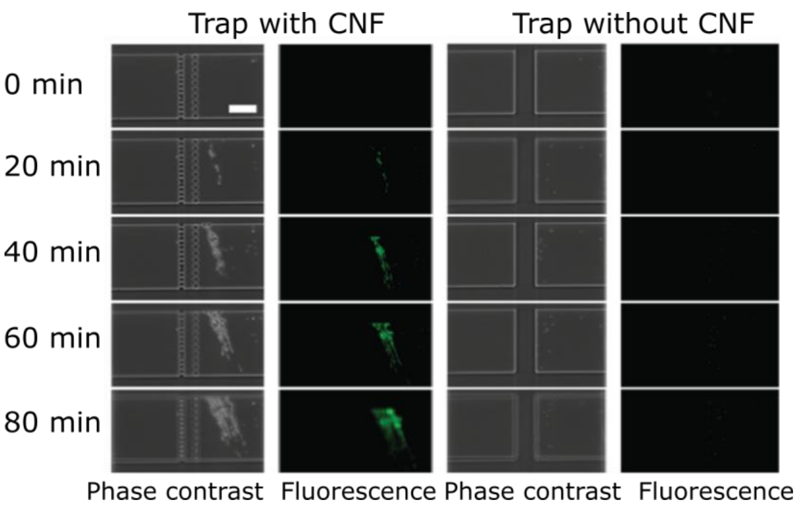

Supplementary Figure 1 Fluorescent and phase contrast microscopy images of microfluidic traps with native CNF hydrogel (two panels on the left) and without CNF hydrogel (two panels on the right) showing the fluorescence accumulation in the CNF hydrogel network due to cationic microplastic particle (PS(ø1µm)) entrapment over time. We took the images during the experiments at given time points, and they are shown as red dots in the manuscript Fig. 1d, where the fluorescence accumulation is shown graphically. Scale bar is 40 µm.

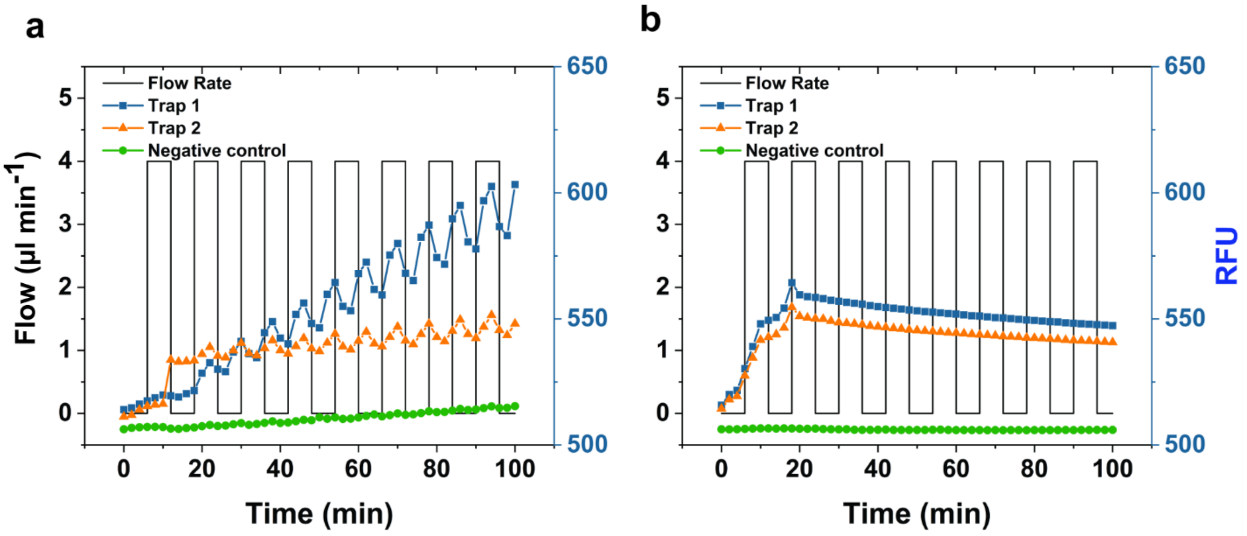

Supplementary Figure 2 Fluorescence accumulation of anionic polystyrene micro- and nanoplastic particles over time by CNF hydrogel network. **a** Data showing accumulation of anionic nanoplastics PS(ø100nm) and **b** data showing accumulation of anionic microplastics PS(ø1µm). Green curves show control trap without CNF hydrogel. The orange and blue curves show parallel experiments with CNF hydrogel in the traps. Source data are provided as a Source Data file.

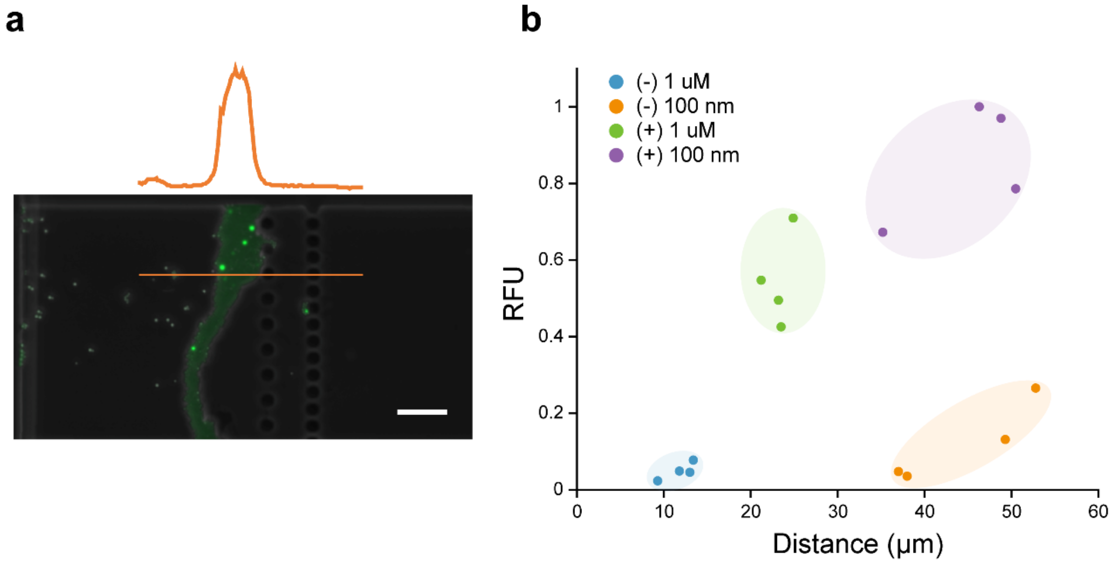

Supplementary Figure 3 Profile analysis of microplastic particles captured by native CNF hydrogel network. **a** Cross-sectional analysis of the CNF hydrogel network during a washing cycle shows the intensity and location of the entangled particles. **b** Cross-section profile analysis of the CNF hydrogel network reveals clear differences between charge (positive and negative) and particle size (nano- and microplastic particle) on the penetration into, and binding affinity towards the CNF hydrogel network. Positively charged particles, both micro and nano-sized display an increased signal, indicating an increased affinity towards the CNF hydrogel, however the nanoplastic particles (PS(ø100nm)) can penetrate further into the hydrogel network than the microplastic particles (PS(ø1µm)). Scalebar in (**a**) is 20 µm. Source data are provided as a Source Data file.

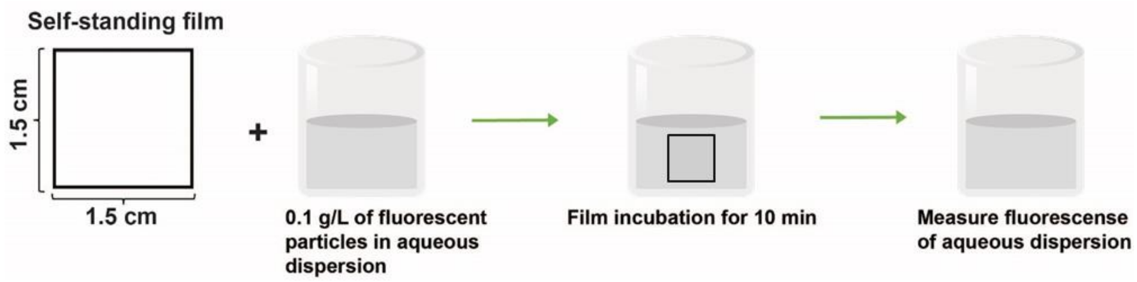

Supplementary Figure 4 The experimental setup for quantitative assessment of the self-standing films' ability to capture plastic particles from aqueous dispersion. T. Loh created the figure using Adobe Illustrator.

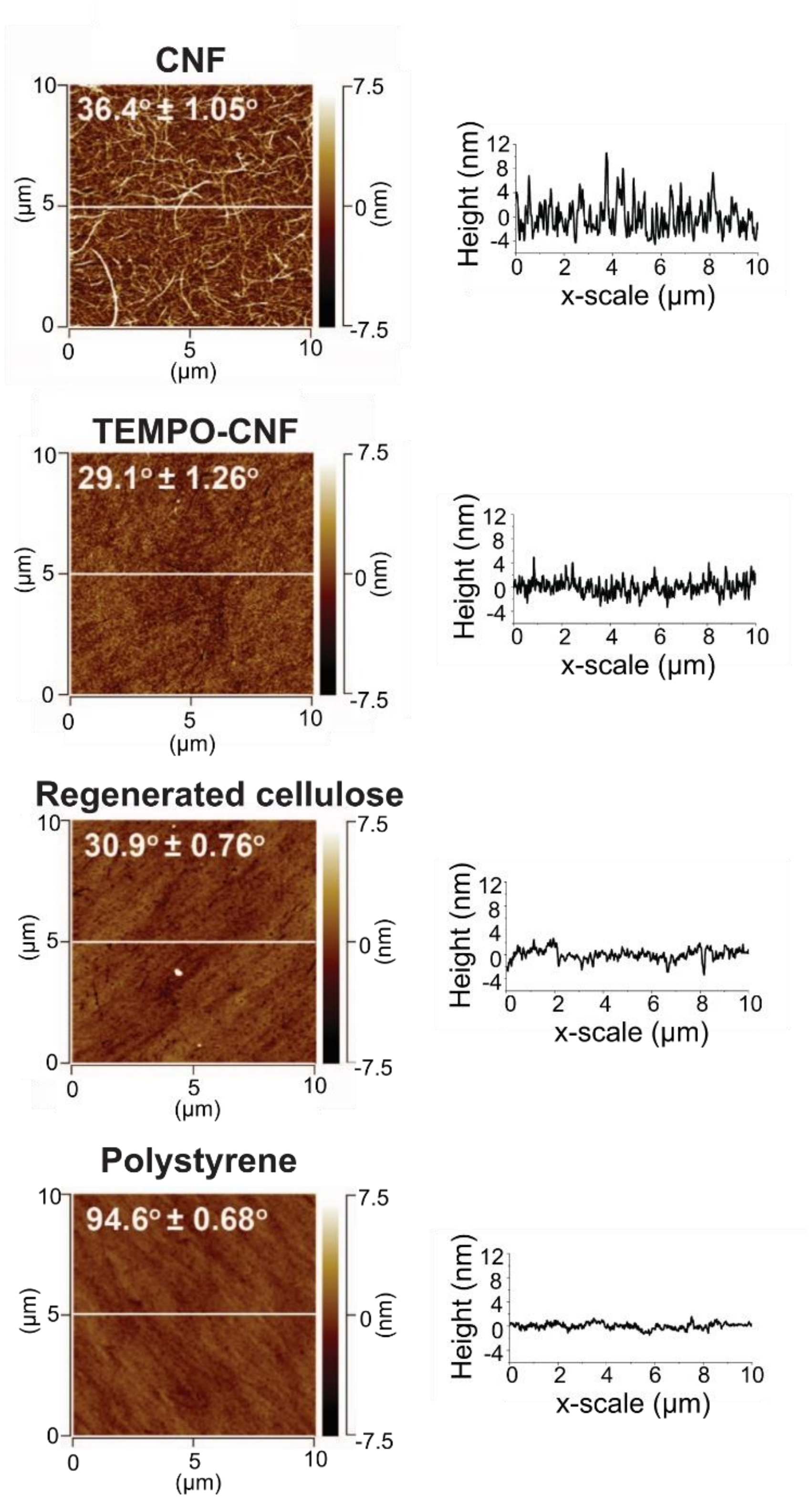

Supplementary Figure 5 Atomic force microscopy (AFM) height images of native cellulose nanofibrils (CNF), TEMPO-oxidized cellulose nanofibrils (TEMPO-CNF), regenerated cellulose (RC), and polystyrene (PS). The white line in the image corresponds to the height profile plot shown beside each image. Static water contact angles for each ultrathin film are shown in the AFM images

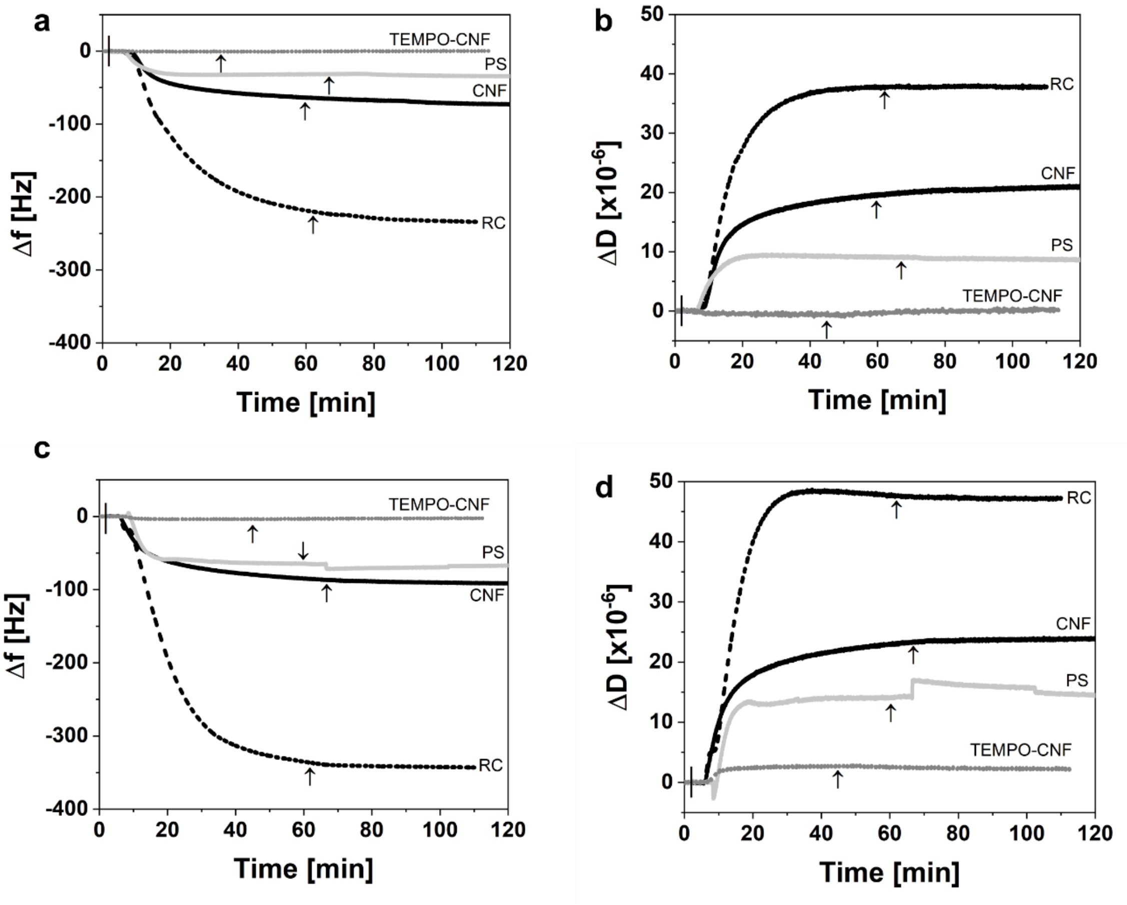

Supplementary Figure 6 Change in frequency (**a,c**) and dissipation (**b,d**) as a function of time as detected by Quartz Crystal Microbalance with Dissipation monitoring (QCM-D). Change in frequency for stabile (**a**) and purified (**c**) nanoplastic particles (PS(ø100nm)). Change in dissipation for stabile (**b**) and purified (**d**) nanoplastic particles (PS(ø100nm)). (f_0 = 5 MHz, n = 5). Arrows indicate the time point when the rinsing with phosphate buffer (10 mM, pH 6.8) was started. The black vertical lines in the beginning of the measurement indicate the time when PS particle injection was started (2min). Source data are provided as a Source Data file.

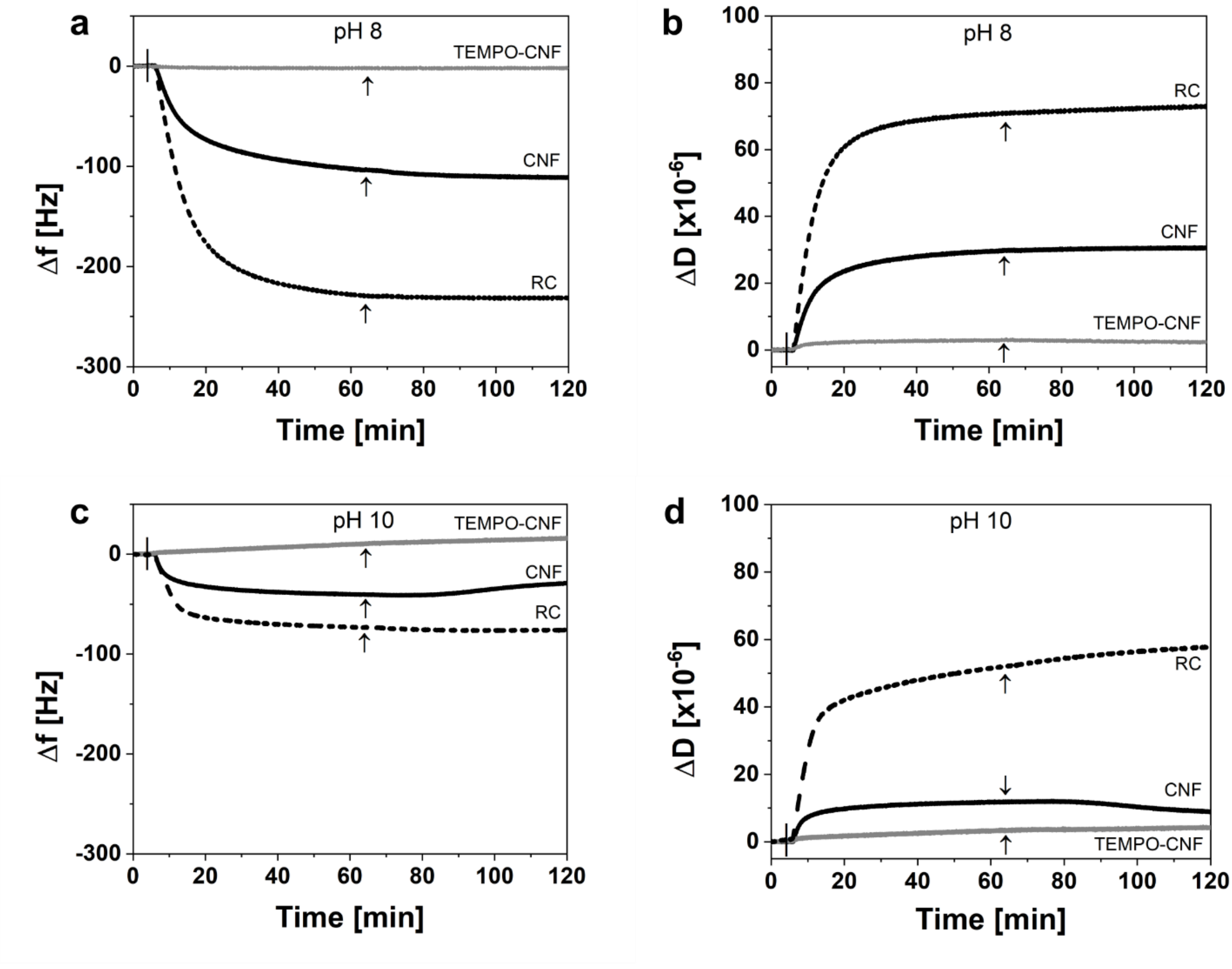

Supplementary Figure 7 Effect of pH on the adsorption. Change in frequency (**a,c**) and dissipation (**b,d**) as a function of time as detected by Quartz Crystal Microbalance with Dissipation monitoring (QCM-D). Change in frequency for stabile PS(ø100nm) in pH 8 (**a**) and pH 10 (**c**). Change in dissipation for stabile PS(ø100nm) in pH 8 (**b**) and pH 10 (**d**). (f_0 = 5 MHz, n = 5). Arrows indicate the time point when the rinsing with buffer (pH 8/pH 10) was started. The black vertical lines in the beginning of the measurement indicate the time when PS particle injection was started (4 min). Source data are provided as a Source Data file.

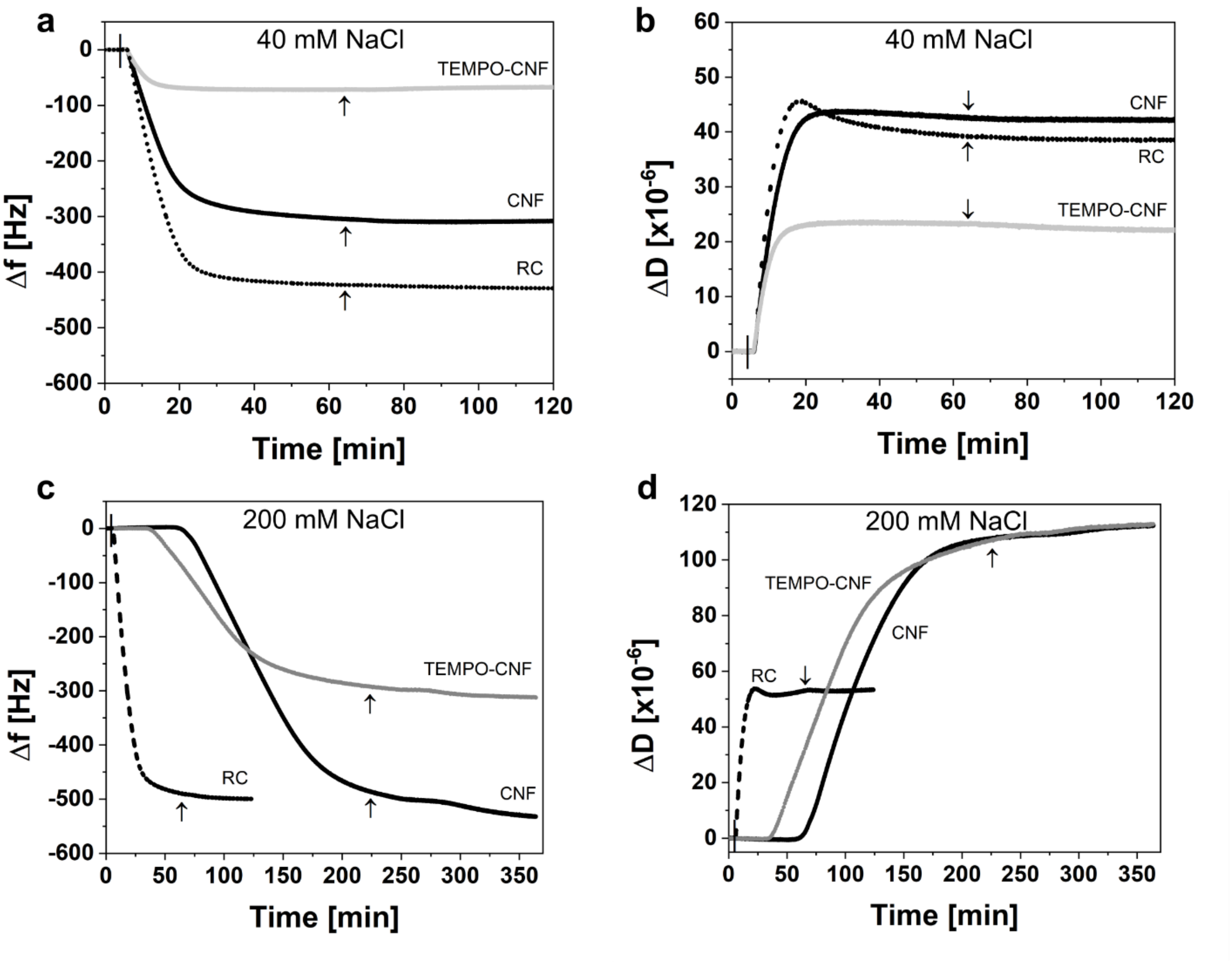

Supplementary Figure 8 Effect of salt concentration on the adsorption. Change in frequency (**a,c**) and dissipation (**b,d**) as a function of time as detected by Quartz Crystal Microbalance with Dissipation monitoring (QCM-D). Change in frequency for stabile nanoplastic particles (PS(ø100nm)) in 40 mM (**a**) and 200 mM (**c**). Change in dissipation for stabile PS(ø100nm) in 40 mM (b) and 200 mM (d). (f_0 = 5 MHz, n = 5). Arrows in indicate the time point when rinsing with phosphate buffer (10 mM, pH 6.8, 40 mM/200 mM NaCl) was started. The black vertical lines in the beginning of the measurement indicate the time when PS particle injection was started (4 min). Source data are provided as a Source Data file.

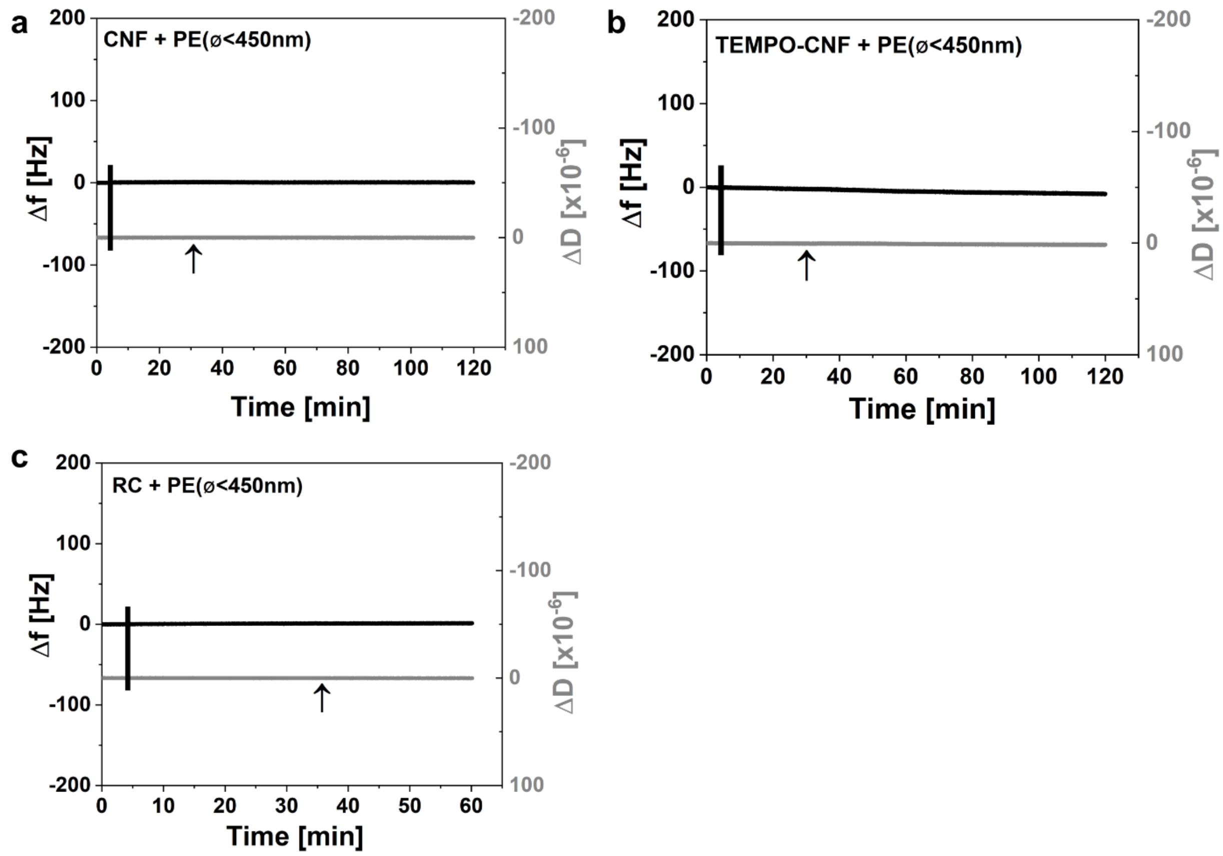

Supplementary Figure 9 Change in frequency and dissipation as a function of time as detected by Quartz Crystal Microbalance with Dissipation monitoring (QCM-D). Change in frequency and dissipation for CNF (**a**), TEMPO-CNF (**b**), and RC (**c**) surfaces when in contact with fractionated polyethylene particles, PE(ø<450 nm). (f_0 = 5 MHz, n = 5). Arrows in indicate the rinsing with phosphate buffer (10 mM, pH 6.8, Tween20 1 wt%) was started. The black vertical lines in the beginning of the measurement indicate the time when PE particle injection was started (4min). Source data are provided as a Source Data file.

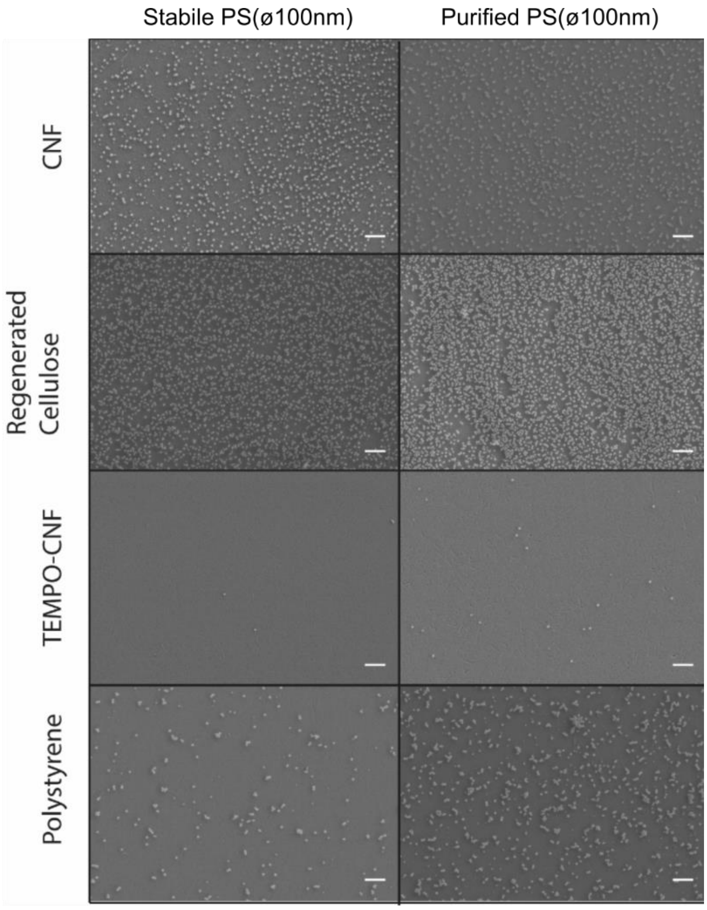

Supplementary Figure 10 Scanning electron microscopy (SEM) images of the QCM-D sensor surfaces after the adsorption experiments. The scale bar is 1 µm.

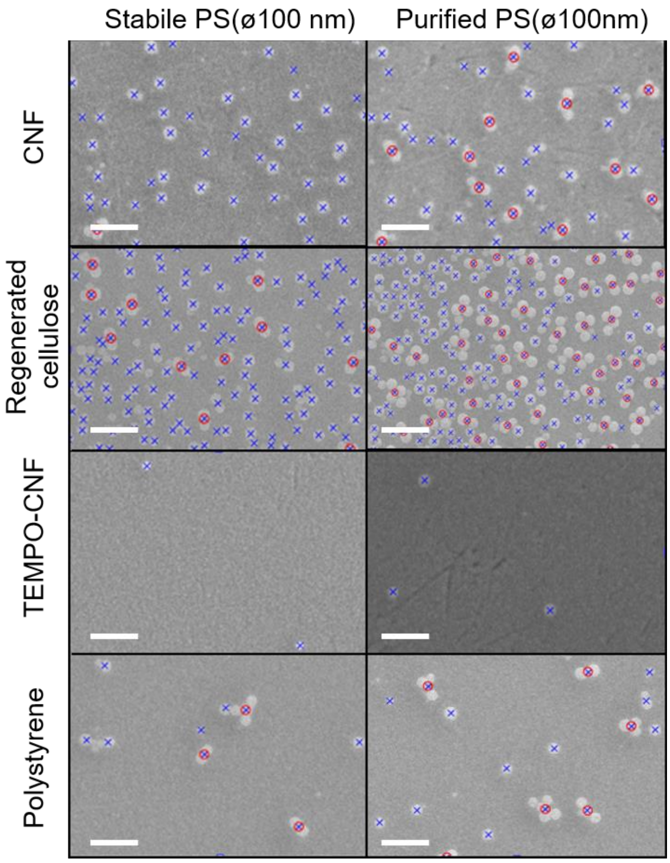

Supplementary Figure 11 Scanning electron microscopy (SEM) images (cropped and zoomed from Supplementary Figure 10) of QCM-D adsorption experiments with identified and calculated nanoparticle objects. Blue cross indicates a single identified nanoplastic particle, and red circle corresponds to an identified cluster with a minimum of three objects. The scale bar in all images is 500 nm.

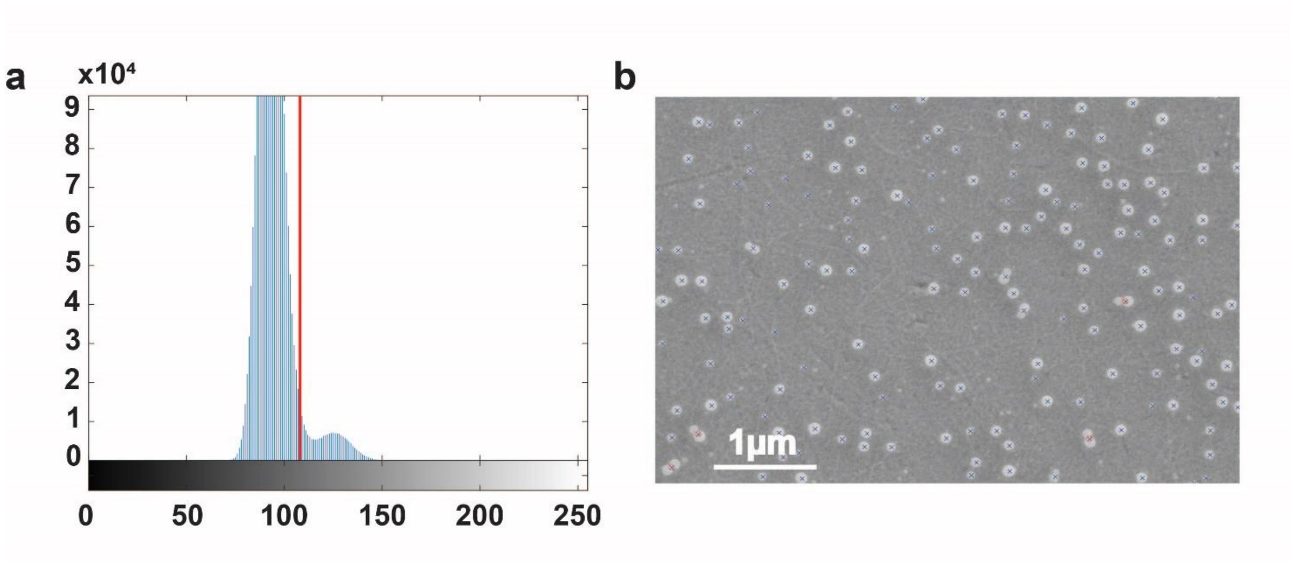

Supplementary Figure 12 a Bimodal histogram of scanning electron microscopy (SEM) image, the threshold used for image segmentation (red vertical line), **b** Nanoplastic particles identified from the image (stabile PS(ø100nm) on native CNF surface).

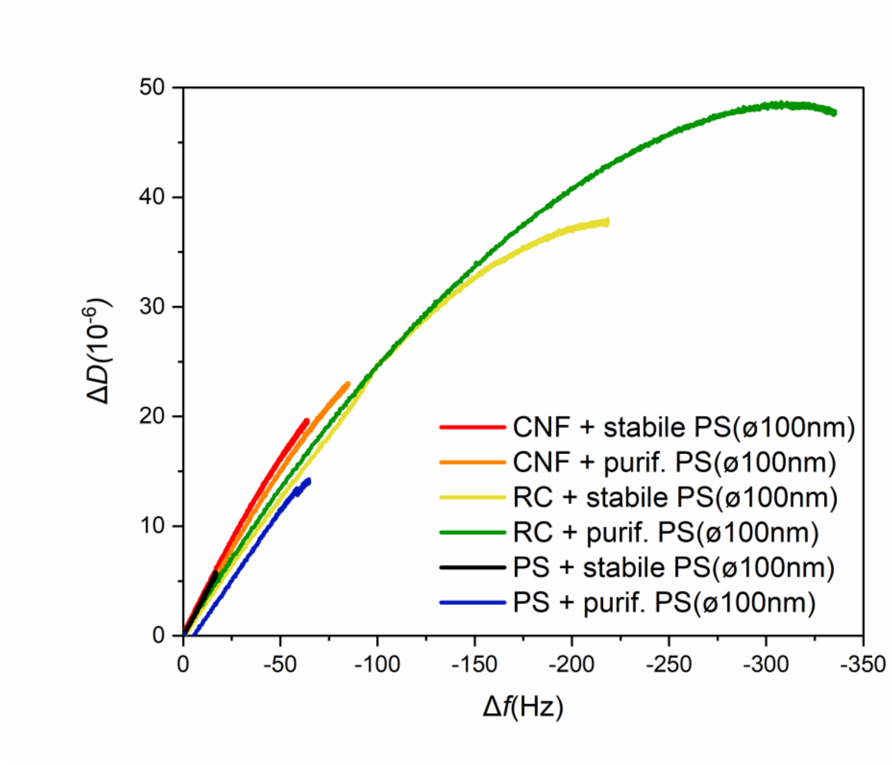

Supplementary Figure 13 Changes in dissipation (ΔD) versus changes in frequency (Δf) for the different nanoplastic particle systems. (f_0 = 5 MHz, n = 5, t = 1h). Source data are provided as a Source Data file.

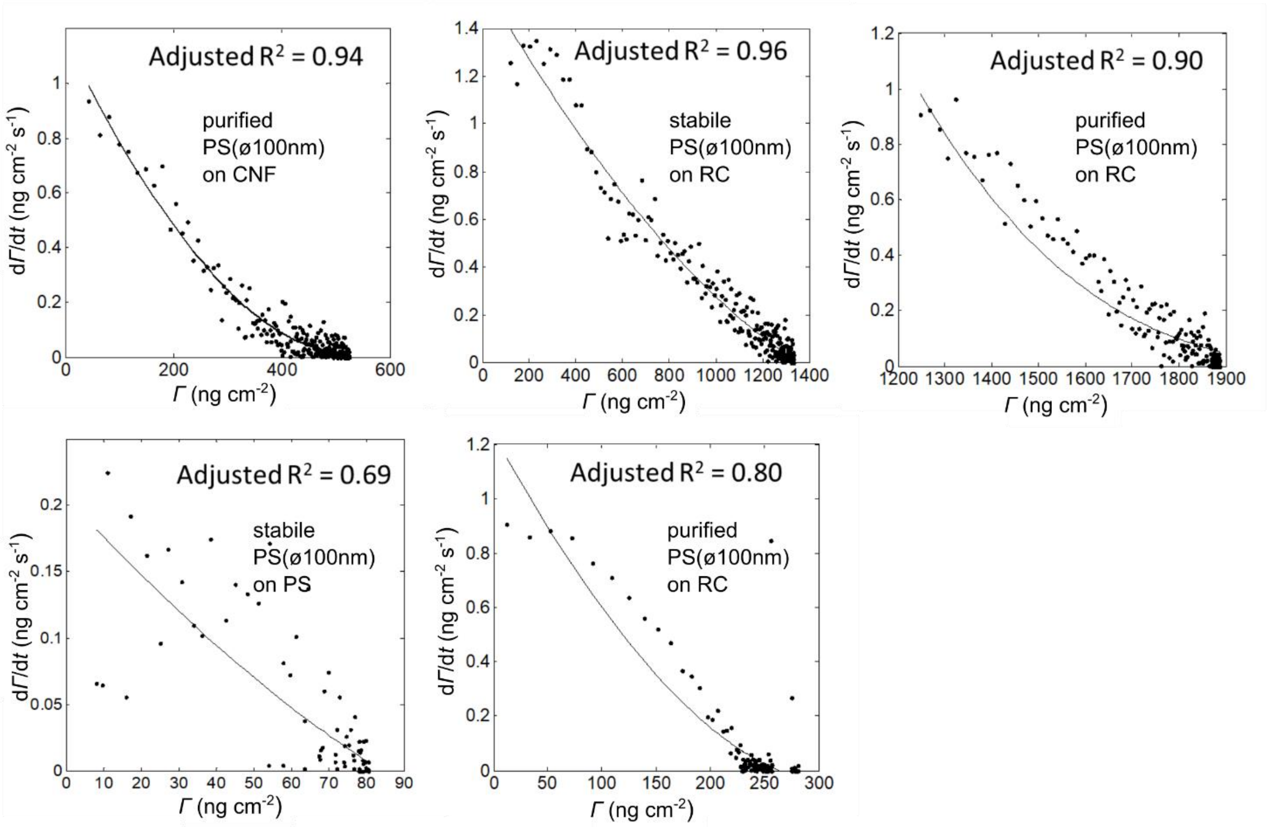

Supplementary Figure 14 Fittings of QCM-D adsorption data of purified and stabile PS(ø100nm) particles on CNF, RC and PS surfaces by the RSA model. Stabile PS(ø100nm) particles on CNF are presented in the manuscript Fig. 3b. Source data are provided as a Source Data file.

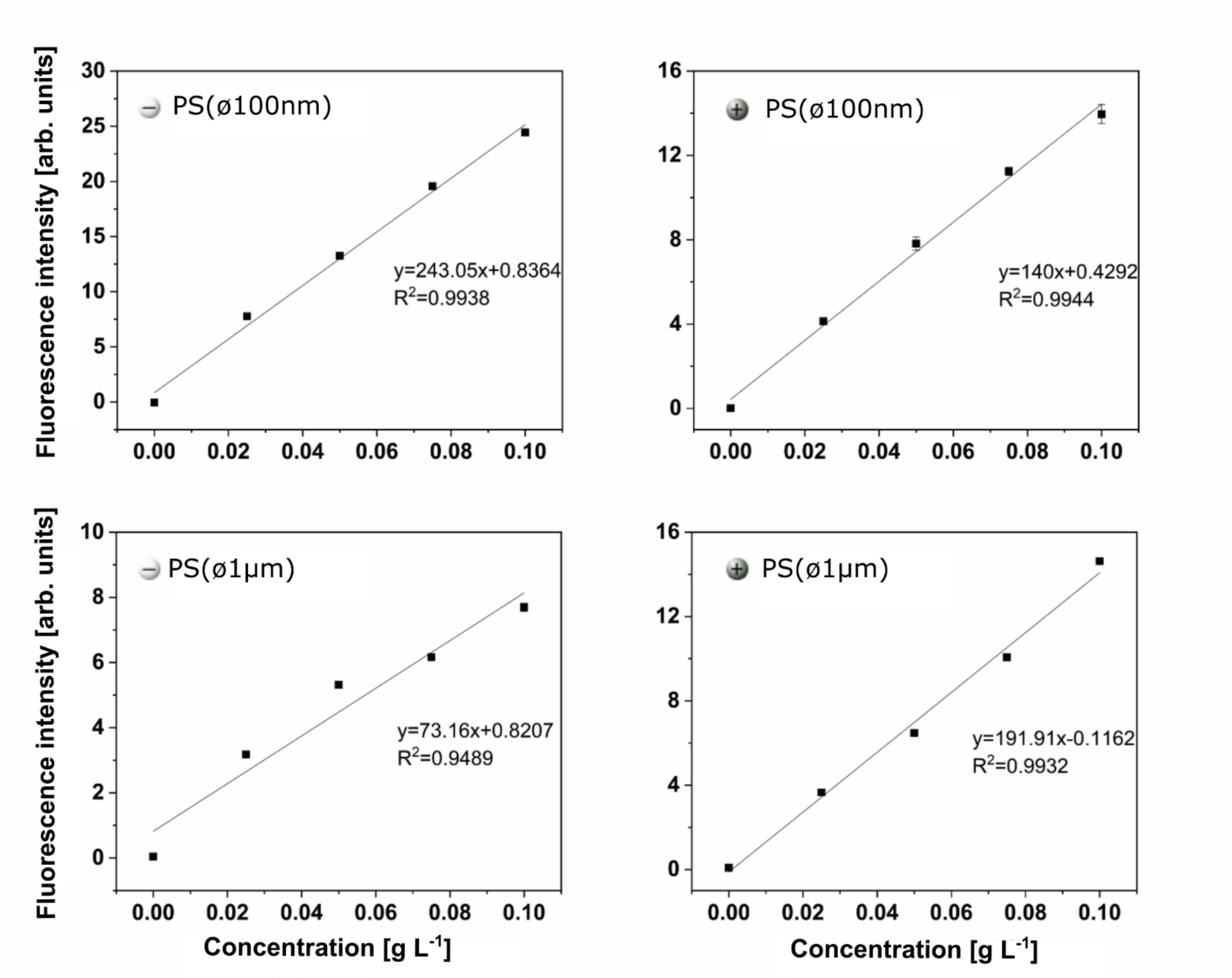

Supplementary Figure 15 Calibration curves for fluorescently labelled nano- and microplastic particles (PS(ø100nm) and PS(ø1µm). Top graphs PS(ø100nm), on the left negatively charged (-) and on the right positively charged (+) particles. Bottom graphs PS(ø1µm), on the left negatively charged (-) and on the right positively charged (+) particles. Error bars indicate mean ± SD. Source data are provided as a Source Data file.

Supplementary Tables

Supplementary Table 1 ζ-potential values of the polystyrene (PS) and polyethylene (PE) particles. (+) stands for cationic and (-) for anionic.

**Particle type**	**ø**	**ζ-potential (mV)[a]**
Fluorescently labelled particles		
PS(ø100nm) (L9902) (-)	100 nm	-53.1
PS(ø100nm) (L9904) (+)	100 nm	16.6
PS(ø1µm) (L4655) (-)	1.0 µm	-47.3
PS(ø1µm) (L9654) (+)	1.0 µm	27.1
PE(ø38-45µm) (UVPMS-BG-1.025)	38-45 µm	-14.5
Non-labelled particles		
stabile PS(ø100nm) (LB1)	100 nm	-52.2
purified PS(ø100nm) (LB1 purified) [b]	100 nm	-44.5
PE(ø200-9900nm) (PENS-0.95)	200-9900	-20.7
PE(ø<450nm) (fractionated PENS-0.95)[c]	< 450 nm	-8.7

[a)] Measurement conditions: 0.05-0.1 gL^{-1} particle solution in phosphate buffer (10 mM, pH 7)
[b)] Purified with the protocol provided by the supplier: dialysis against Milli-Q water with 1000 kDa MWCO dialysis membrane (Biotech CE Membrane Dialysis Trial Kit)
[c)] Fractionation with PVDF membrane (Millipore, 0.45 µm)

Supplementary Table 2 The number of cationic (+) and anionic (-) 100nm and 1.0 µm PS particles entrapped by self-standing films. Source data are provided as a Source Data file.

Film	Polystyrene (PS) particles	# of PS particles/mm^2
CNF	PS(ø1µm) (+)	$2.51\times10^5 \pm 2.93\times10^4$
	PS(ø1µm) (-)	$0.836\times10^5 \pm 1.33\times10^4$
	PS(ø100nm) (+)	$1.04\times10^8 \pm 5.93\times10^7$
	PS(ø100nm) (-)	$0.529\times10^8 \pm 1.61\times10^7$
TEMPO-CNF	PS(ø1µm) (+)	$3.27\times10^5 \pm 2.93\times10^4$
	PS(ø1µm) (-)	$1.03\times10^5 \pm 2.77\times10^4$
	PS(ø100nm) (+)	$1.67\times10^8 \pm 2.48\times10^7$
	PS(ø100nm) (-)	$2.45\times10^8 \pm 3.60\times10^7$
Polystyrene	PS(ø1µm) (+)	$1.14\times10^5 \pm 0.993\times10^4$
	PS(ø1µm) (-)	$0.589\times10^5 \pm 1.90\times10^4$
	PS(ø100nm) (+)	$1.34\times10^8 \pm 2.50\times10^7$
	PS(ø100nm) (-)	$0.446\times10^8 \pm 1.37\times10^7$
Regenerated Cellulose	PS(ø1µm) (+)	$1.69\times10^5 \pm 6.82\times10^4$
	PS(ø1µm) (-)	$0.877\times10^5 \pm 1.80\times10^4$
	PS(ø100nm) (+)	$1.14\times10^8 \pm 1.43\times10^7$
	PS(ø100nm) (-)	$1.44\times10^8 \pm 1.71\times10^7$

Supplementary Table 3. The area of self-standing films needed to recover all cationic (+) and anionic (-) 100 nm and 1.0 µm PS particles from the 0.1 g L^{-1} solutions. Source data are provided as a Source Data file.

Film	Polystyrene (PS) particles	Area (cm^2) needed to clear all particles[a)]
CNF	PS(ø1µm) (+)	30
	PS(ø1µm) (-)	85
	PS(ø100nm) (+)	70
	PS(ø100nm) (-)	140
TEMPO-CNF	PS(ø1µm) (+)	20
	PS(ø1µm) (-)	70
	PS(ø100nm) (+)	45
	PS(ø100nm) (-)	30
Polystyrene	PS(ø1µm) (+)	65
	PS(ø1µm) (-)	125
	PS(ø100nm) (+)	55
	PS(ø100nm) (-)	165
Regenerated Cellulose	PS(ø1µm) (+)	45
	PS(ø1µm) (-)	85
	PS(ø100nm) (+)	65
	PS(ø100nm) (-)	50

[a)] Amount of PS(ø100nm) and PS(ø1µm) in solution are calculated based on the solution fluorescence before film immersion and the standard curves for the particles. The amount of PS(ø100nm) in a 0.1 g L^{-1} solution is ~730G and PS(ø1µm) ~725M.

The maximum amount of particles that can be accommodated on different planar surfaces depends on the film's thickness and how the particles can penetrate inside the film. The self-standing films used in this study are very different in structure; they are made of different materials, two are colloidal (CNF, TEMPO-CNF), and two are polymeric (RC, PS). CNF and TEMPO-CNF have a large surface area compared to RC and PS, and they form porous films allowing particles to /enter inside the film structure/penetrate the film structure. The cellulose materials (CNF, TEMPO-CNF, and RC) swell significantly in water, affecting their thickness and thus volume. Both experiments and computer simulations have shown that the maximum volume fraction of randomly packed equal-sized hard spheres is 64% (Bernal close-packing limit).[1] However, because the volumes of the self-standing films are different, time-dependent, and challenging to determine, it is impossible

to make comparisons concerning the theoretical maximum number of particles per unit area of the film. Thus, the performance of the different films was assessed by comparing the area of each film needed to capture all particles present in the solution (Supplementary Table 3).

Supplementary Table 4. The area of self-standing films needed to recover all PE particles (øPE(38-45µm) from 0.1 g L^{-1} solutions. Source data are provided as a Source Data file.

Film	Change in fluorescence intensity (%)	PE mass captured (µg cm^{-2})	Area (cm^2) needed to clear all PE particles [a)]
CNF	-13.1	26	16
TEMPO-CNF	-15.5	24	17

[a)] Number of particles could not be quantified in a similar fashion as for PS particles, since the PE particle dispersion is heterogeneous with respect to the particle size distribution. Thus, we calculated only the mass recovered based on the solution fluorescence before and after immersion of the film (change in fluorescence intensity directly proportional to the concentration and mass recovered). The mass of particles in a 0.1 g L^{-1} solution is 0.4 g.

Supplementary Table 5. The number of stabile (S) and purified (P) 100 nm PS particles (PS(ø100nm)) adsorbed on various substrates compared to the theoretical maximum number that can adsorb on the surface. Source data are provided as a Source Data file.

Film	Polystyrene (PS) particles	# of PS particles mm^{-2} [a)]
CNF	PS(ø100nm) (P)	$9.96\times10^6 \pm 1.60\times10^6$
	PS(ø100nm) (S)	$7.97\times10^6 \pm 1.30\times10^6$
TEMPO-CNF	PS(ø100nm) (P)	$16.5\times10^4 \pm 1.51\times10^4$
	PS(ø100nm) (S)	$1.33\times10^4 \pm 0.87\times10^4$
PS	PS(ø100nm) (P)	$5.35\times10^6 \pm 2.08\times10^6$
	PS(ø100nm) (S)	$1.53\times10^6 \pm 0.164\times10^6$
RC	PS(ø100nm) (P)	$35.9\times10^6 \pm 1.38\times10^6$
	PS(ø100nm) (S)	$25.3\times10^6 \pm 1.79\times10^6$

[a)] # of PS particles mm^{-2} is the number of particles per unit area (dN/dt) after the adsorption process gained by applying the image analysis.

Supplementary References

1. Baranau, V. & Tallarek, U. Random-close packing limits for monodisperse and polydisperse hard spheres. *Soft Matter* **10**, 3826–3841 (2014).
