# Supplementary material for: Capturing colloidal nano- and microplastics with plant-based nanocellulose networks
Source: Nat Commun. 2022 Apr 5;13:1814. doi: 10.1038/s41467-022-29446-7 (PMC8983699; doi:10.1038/s41467-022-29446-7)
Supplement: Supplementary file 3 — Description of Additional Supplementary Files [file 41467_2022_29446_MOESM3_ESM.pdf]

Description of Additional Supplementary Files

Capturing colloidal nano- and microplastics with plant-based nanocellulose networks

Ilona Leppänen[1], Timo Lappalainen[1], Tia Lohtander[1,2], Christopher Jonkergouw[3], Suvi Arola[1*] and Tekla Tammelin[1*]

File Name: Supplementary Video 1

Description: Accumulation of fluorescently labelled polystyrene particles (PS(ø1µm)) in microfluidic traps containing nanocellulose (native CNF) hydrogel. The capture of particles is verified using the microfluidic set-up and fluorescent imaging.
